# Attitudes of Austrian Psychotherapists Towards Process and Outcome Monitoring

**DOI:** 10.1007/s10488-018-0862-1

**Published:** 2018-03-08

**Authors:** Tim Kaiser, Lisa Schmutzhart, Anton-Rupert Laireiter

**Affiliations:** Department of Psychology, Psychotherapy Research Group, University of Salzburg, Hellbrunnerstrasse 34, 5020 Salzburg, Austria

**Keywords:** Monitoring attitude, Validation, Outcome monitoring, Process monitoring, Outcome Measurement Questionnaire

## Abstract

While monitoring systems in psychotherapy have become more common, little is known about the attitudes that mental health practitioners have towards these systems. In an online survey among 111 Austrian psychotherapists and trainees, attitudes towards therapy monitoring were measured. A well-validated questionnaire measuring attitudes towards outcome monitoring, the Outcome Measurement Questionnaire, was used. Clinicians’ theoretical orientations as well as previous knowledge and experience with monitoring systems were associated with positive attitudes towards monitoring. Possible factors that may have led to these findings, like the views of different theoretical orientations or obstacles in Austrian public health care, are discussed.

## Introduction

Even though large treatment effects were found for various forms of psychotherapy, a stable number of patients do not improve or even deteriorate in the course of treatment. Additionally, the average rate of patients who prematurely drop out of treatment was estimated at 47% in an early meta-analysis (Wierzbicki and Pekarik 1993) and 20% in more recent ones (Swift et al. 2017). As shown by Hatfield et al. (2010), 70% of deteriorations of patients remain undocumented (and probably unnoticed), indicating that psychotherapists have great difficulties in detecting these undesirable changes. It was concluded that additional assessment tools are necessary to improve outcomes of psychotherapy. Various methods of monitoring success of ongoing psychotherapies have been developed (see Drapeau 2012 for an overview) after initial suggestions and pioneering work by Howard et al. (1996). On a regular basis (e.g. before or after therapy sessions), patients fill out feedback questionnaires that provide therapists with insights into treatment progress and related constructs. The instruments used are quite diverse and range from very short scales to more comprehensive instruments. For example, the the “Partners of Change Outcome Management System” (PCOMS, Duncan 2012) employs two scales with four items used to rate treatment outcomes and the therapeutic alliance. Despite its brevity, the PCOMS was shown to reduce the number of patients who deteriorate by 50% (Lambert and Shimokawa 2011). Longer, more comprehensive instruments like the Outcome Questionnaire (OQ-45, Lambert and Finch 1999) or the CORE-OM (Evans et al. 2002) show similar effects. Constructs measured by outcome monitoring tools can include symptoms of psychopathology, the patient’s social relationships or their social role, but also the quality of the therapeutic alliance, treatment motivation, etc. Additionally, some outcome monitoring systems interpret the scores of feedback questionnaires and derive clinical advice for therapists. These advices originally included addressing broad domains relevant for treatment success, like treatment motivation or the therapeutic relationship, but as outcome monitoring systems become more and more advanced, specific interventions and relevant material for in-session use, like worksheets or exercises, are directly provided by the system (e.g. Lambert 2015). A lot of studies demonstrated that these monitoring approaches enhance treatment outcomes and prevent negative outcomes (Shimokawa et al. 2010).

Like psychotherapy research is commonly divided into process and outcome research (Gelo et al. 2014), monitoring does not necessarily have to focus on outcome alone, but on change processes occurring in between therapy sessions that remain undetected because the vast majority of outcome monitoring methods are assessed in-session. Real-time monitoring of change processes is justified by an increasing body of evidence that processes that occur in between sessions are highly relevant for successful treatment (Stewart and Schröder 2015). Hence, information on these so-called “intersession processes” (Orlinsky et al. 1993) could offer clinicians valuable insights on how their patients process the current therapies. Due to technological limitations, most process studies on change processes have focused on retrospective questionnaires and interviews, investigating interindividual variation of process variables. However, following the arguments presented by Molenaar (2013), this approach can hardly be classified as “process research”, as psychological processes neither have constant characteristics over time, nor are statistical models derived from interindividual variation valid for processes in individuals. Instead, process data has to be sampled with high frequency from individuals. The emergence of modern technologies like Internet-enabled mobile devices in mental health contexts gave rise to research literature on possible applications (e.g. Ben-Zeev et al. 2013; Torous and Powell 2015) in assessment and treatment of mental disorders. Using mobile devices, it became possible to monitor processes relevant to psychotherapeutic interventions with the high frequency necessary to generate valid data sets, i.e. once or even multiple times per day. This enables practitioners to gather information on change processes in patients while they evolve, i.e. in real time, or even to plan interventions before treatment onset (Fernandez et al. 2017; Fisher and Boswell 2016). On the client side, process monitoring allows for an increased intensity of reflection of the current treatment, which is in turn speculated to lead to increased self-efficacy, therapy motivation and even emotional competence (Schiepek and Aichhorn 2013), but these assumptions have not yet been investigated empirically.

Concerning technology for high-frequency process monitoring, an important contribution has been made with the introduction of the “Synergetic Navigation System” by Schiepek and Strunk (2010). This software package is able to send out daily questionnaires that have been developed specifically for process monitoring, like the Therapy Process Questionnaire (TPB, Schiepek et al. 2012), but also outcome-related measures and other instruments. Patients are reminded via SMS or E-Mail messages and are able to access questionnaires via their mobile devices or personal computers. Data on feasibility and patient compliance is promising (Schiepek et al. 2016) and data derived from process monitoring has been shown to be predictive of treatment success (Schiepek et al. 2014).

The DynAMo software package for process monitoring was introduced by Kaiser and Laireiter (2017). It offers an initial implementation of open-source tools that can be used for process monitoring. Similar to the SNS, this software can be set up to send out regularly-timed online questionnaires to patient’s own devices. The obtained data can be viewed by researchers and practitioners to get real-time feedback. Currently, DynAMo is undergoing testing for various applications in single-case and group studies to explore its utility for various tasks in psychotherapy. This includes personalized feedback for therapeutic processes and symptoms, process monitoring in private practice, and prediction of outcomes based on process data.

The implementation of monitoring systems in mental health institutions is a long-standing concern. Introducing new methods of assessment or even new technologies can conflict as well with daily routines in clinics as with convictions from practitioners’ psychotherapy training. This could be one of the reasons why it is common that mental health practitioners are skeptical of monitoring or even reject using it entirely. Since outcome monitoring became mandatory in Australia, reactions of clinicians to these technologies have been mixed, as interviewing studies have shown (Callaly et al. 2006). Attitude towards outcome monitoring before it was implemented was predictive of actual active use of monitoring, which in turn led to the positive effects monitoring has for “off track” cases in psychotherapy (De Jong et al. 2012).

This motivated researchers to investigate the issues and to identify possible barriers. A systematic review of qualitative studies by Boyce et al. (2014) showed various concerns of clinicians that can be grouped into four themes. First, practical concerns were mentioned, relating mainly to an increased workload introduced by monitoring, usage difficulties due to lack of user-friendliness, or lack of appropriate training. Physicians and nurses also often lack statistical knowledge, leading to issues in data interpretation. The second theme referred to concerns about the purpose of data collection and potential misuse, as well as a lack of openness to feedback. Additionally, some clinicians doubted the clinical utility of the data when it comes to capturing what is really relevant to a successful treatment or to professional reflection. Some of these issues were addressed by Boswell et al. (2015), who provided guidelines for stakeholders with the intention to implement monitoring. More low-threshold and practical reflections were provided by De Jong (2016), who identified difficulties in dealing with negative feedback obtained through monitoring systems as an important barrier in implementation. If negative feedback from monitoring data contradicts a clinician’s positive beliefs about a patient’s progress, this can be a distressing experience. Practitioners may attribute the lack of progress to client factors in order to protect their professional self-esteem or attribute negative outcomes internally, possibly leading to burn-out. Thus, it is important to address these attributions during training and to emphasize that feedback is not fit to compare the success of individual therapists. Trainers should instead focus on the use of monitoring systems as a tool for identifying cases at risk for deterioration, which is likely to be aligned with therapists’ professional goals.

A recent notable example for effectively fostering use and perceived clinical utility of outcome monitoring, the “National Routine Outcome Monitoring Quality Improvement Collaborative” (National ROM QIC) was recently evaluated by Metz et al. (2017), showing promising results. The initiative included conferences, training programs for practitioners and booster sessions for exchange. This large, government-financed initiative contributed to a vast increase in use of monitoring systems as well as a much higher perceived clinical utility (effect sizes of d = .99 –1.25). This stresses the importance of coordinated efforts in implementation of monitoring.

Despite the empirical evidence on its beneficial effects, and with some notable exceptions, a broad implementation of outcome monitoring in German-speaking countries still did not take place yet. As Puschner et al. (2015) concluded in their overview of case studies, this is likely to be caused by the particularities of the fragmented German health care system, especially by the lack of central coordination. The same could be concluded for Austria, where no broad attempts to implement monitoring have been made up to now. In Austria, the field of psychotherapy is highly diverse, as different professions work in the field of psycho-social treatment supply. Also, therapeutic orientations with little interest in implementing monitoring are highly influential.

For the relatively new field of process monitoring, systematic studies are rare and only available as “gray literature”. One qualitative survey on the implementation of process monitoring in a German clinic (Eschenbacher 2015) identified issues similar to those in outcome monitoring and it seems valid to assume that the concerns about low-frequency outcome monitoring increase when process monitoring is considered, as it is methodologically more challenging, requires additional training and is potentially more time consuming.

Quantitative data on the attitude of clinicians towards process and outcome monitoring is also scarce. First attempts to assess and even improve this attitude have been made by Willis et al. (2009), leading to a training program and a questionnaire to measure monitoring attitudes (the Outcome Measurement Questionnaire, OMQ), allowing researchers to generate comparable data sets. While attitudes toward outcome monitoring were already mainly positive in the baseline measure, it was shown to improve after a training workshop on this subject. The resulting questionnaire was validated with a larger sample by Smits et al. (2015), who also translated the OMQ to the Flemish language. In this study, attitude toward outcome monitoring was better for practitioners with a higher level of education and psychotherapeutic training. Also, psychotherapists in private practice had significantly more positive attitudes compared to practitioners working in inpatient and subsidized outpatient settings. The effect of training programs on outcome monitoring attitude was confirmed by Edbrooke-Childs et al. (2016), who used the OMQ in a training program for outcome measures in child mental health. Both attitude and self-efficacy concerning outcome monitoring improved following 1 or 3-day workshops that were designed to overcome personal barriers to using outcome monitoring as well as practical and theoretical training in the use of monitoring systems. All studies found good reliability, validity and sensitivity to change for the OMQ.

### Aims and Objectives

The goal of this study was to gather information on the attitudes of clinicians towards process and outcome monitoring in Austria. Also, variables that possibly influence this attitude were investigated. To achieve this, the OMQ was translated to German and subjected to item and factor analysis. Additionally, a short scale measuring the attitude towards process monitoring (Process Monitoring Questionnaire, PMQ) was developed.

## Methods

### Data Collection

The data collection period ran from May 16th to July 10th, 2017. An online survey was conducted using the SoSciSurvey platform (Leiner 2014). Clinicians were contacted publicly available E-mail addresses from the Austrian psychotherapy association (ÖBVP), the Salzburg association for cognitive-behavioral therapy (AVM), the institute for synergetics and psychotherapy research of the Salzburg Paracelsus medical school and the counseling center of the University of Salzburg. Personalized salutations were used to increase response rate. E-Mails included a request to distribute the survey among colleagues. Possible participants following the study’s URL were greeted with an introduction page containing general information. This included the goal of the study (i.e. assessing the attitude of psychotherapists towards process and outcome monitoring), expected duration of the study, and the procedure of the following questionnaire. Potential participants were informed that data would be used for research purposes. After deciding to participate, subjects were presented with a brief information text on process and outcome monitoring. The text was designed so that it contains vital information on both process and outcome monitoring that is relevant to practitioners, while being concise enough not to overstrain the participants. Participants were instructed to read the text carefully as the information presented is relevant for answering the following questionnaire. A translation of the information text presented follows.

The terms “process monitoring” and “outcome monitoring” refer to the continuous monitoring of effects in psychotherapy as well as to monitoring processes and trajectories of change in psychotherapy.

In regularly timed intervals, data including (but not limited to) patients’ attitudes towards treatment, symptom severity, affectivity, the quality of patients’ interpersonal relationships or motivation to change are obtained based on self-reports from clients. This enables practitioners to get a prompt feedback on the course and effects of treatment, and to detect possible deteriorations.

In process monitoring, patterns of change can be detected by direct systematic assessment of the therapeutic process using online questionnaires and mobile apps. This is achieved by conducting fine-grained and mostly daily (real-time) collection of information on how clients process their therapies. The resulting information is usually discussed with the client in feedback sessions.

In outcome monitoring, information relevant for treatment success (e.g. symptom severity, quality of the working alliance, treatment motivation) is collected mostly in weekly intervals. By utilizing normative data, a specific course of treatment can be compared to courses of clients with a similar diagnostic profile. This enables the practitioner to determine if a patient’s progress is “on track”.

Over the last years, monitoring systems integrating assessment, analysis and visualization of data were developed to provide practitioners with optimal feedback on change processes. In feedback sessions, the data collected can be discussed with the patient.

The text was followed by two questionnaires measuring attitudes towards outcome and process monitoring. Finally, a demographic questionnaire assessed gender, age, nationality, level of education, and university degrees. Clinician characteristics were assessed, including theoretical orientation, years of clinical experience and previous experience with monitoring systems. Optionally, participants could provide their view on advantages and disadvantages of monitoring in free-form text fields. The dataset was anonymized and responses were impossible to trace back to individual participants. Technical information that could compromise anonymity (IP addresses, web browser fingerprints) was not collected and no HTTP cookies were set. During data collection, access to the data set was limited to the first and second author of this study.

Due to the anonymity of the survey, it was unknown to the investigators whether a therapist already answered, so no reminder messages were sent. No incentives were given to participants. A contact mail address was given to the participants after completion of the survey, providing them with a means of contacting the authors of this study in case of questions or remarks. Contacting the authors did not enable them to link a specific sender address to answers to the survey.

### Participants

After contacting 1212 psychotherapists, 241 opened the survey URL (response rate of 20%), 130 closed the survey page without proceeding beyond the greeting text, so that 111 participants who completed the survey remained (9.16% retention rate). Age, gender and clinical experience data is summarized in Table 1. Regarding theoretical orientation, 40 participants followed humanistic and existential orientations, 27 a cognitive-behavioral one, 24 psychodynamic approaches and 20 a systemic orientation. 25 participants were still in training. 85 participants had no previous practical experience with monitoring systems and 26 indicated that they had. From these 26 participants, six were currently using a monitoring system and three took part in training seminars for those.

**Table 1:** Age and years of clinical experience of participants

Participants	n	Mean age (years)	SD	Range (years)
Total	111	51.39	11.75	27–79
Women	71	48.92	10.15	28–67
Men	40	55.78	13.18	27–79

Participants	n	Mean years of clinical experience	SD	Range (years)
Total	109	15.32	12.73	0–45
Women	69	11.72	10.48	0–38
Men	40	21.45	13.97	0–45

Clinical experience was not provided by two female participants

### Instruments and Translation Procedure

The OMQ by Willis et al. (2009) was developed for assessing attitudes towards routine outcome monitoring in a mental health context. In previous studies, the OMQ reached satisfactory internal consistency (Cronbach’s alpha ranging from .79 to .89) and an adequate factor structure (Smits et al. 2015). However, a German language version of this instrument was not available at the beginning of this period. Thus, the authors decided to translate this instrument. The translation was conducted according to the Guidelines from the European Social Survey Programme (European Social Survey 2016). In the first step, OMQ items were translated independently by two bilingual assistants. They were instructed to keep translations close to the original, whilst providing adequate comprehensibility and fluency. The translations were then compared and combined to a preliminary version. This version was revised again by the authors together with a bilingual native English speaker to identify linguistic weaknesses in the translation. The OMQ consists of 23 items that are rated on a six-point Likert scale, ranging from “Strongly disagree” (1) to “Strongly agree” (6) (see “Appendix 1: OMQ Items**”** for details). In the original version, the authors proposed two rationally constructed subscales named “Openness to feedback” and “Monitoring attitude”. These subscales could not be confirmed in a factor analysis by Smits et al. (2015), who instead found a factor solution with one factor including positively coded items and one method factor including reverse-scored items to be of best fit. The first factor was correlated almost perfectly with OMQ scores (r = .97), justifying the use of a total sum score of the OMQ to measure attitudes towards monitoring (details in “Appendix 2: PMQ Items”).

Because the OMQ only includes items on routine outcome monitoring, which not necessarily is administered with high temporal frequency, a short questionnaire consisting of eight items on daily process monitoring was constructed. Item formulations for this “Process Monitoring Questionnaire” (PMQ) were designed to match the content of OMQ items addressing general attitude and intention of use versus criticism. Other items were related to putative specific effects of high-frequency process monitoring like increased self-reflection, improved therapeutic alliance or facilitating detection of possible deterioration, as proposed by Schiepek et al. (2016).

### Data Analysis

Descriptive statistics, *t* tests, ANOVA, correlations and reliability calculations were performed using the R programming language (R Core Team 2016). Two-tailed independent *t* tests were conducted to compare mean scores of this sample to three other samples (Edbrooke-Childs et al. 2016; Smits et al. 2015; Willis et al. 2009). After applying Bonferroni-correction for three simultaneous comparisons, the critical p value was set to p = .017. With the obtained sample size of 111, an effect of r = .262 (r^2^ = .068 or d = 0.534) can be detected with a power of .80.

An ANOVA was conducted to examine main and interaction effects of clinician characteristics (gender, past experiences with monitoring systems, theoretical orientation) on OMQ and PMQ scores. Omega-squared (${\omega ^2}$) was used as an effect size measure for ANOVAs, as it is a less biased alternative to the more common eta-squared (${\eta ^2}$) (Okada 2013). Interpretations of effect sizes are given according to meta-analytically derived guidelines by Gignac and Szodorai (2016). According to these guidelines, effects of r < .16 or lower are considered low, r < .25 is considered medium and r > = .37 large. Similar to ${\eta ^2}$, the magnitude of ${\omega ^2}$ can be interpreted like r^2^ (Cohen 1988).

To examine the relationship of interval-scaled variables (age, years of clinical experience) with OMQ and PMQ scores, Pearson correlations were calculated. The critical p-value was set to p = .025 after correcting for two simultaneous correlations. Cronbach’s alpha was calculated to evaluate the reliability of the OMQ and PMQ scales.

Participants were invited to provide free text responses to two open questions concerning advantages and disadvantages of monitoring. These answers were compiled to separate text files (advantages and disadvantages) and analyzed with Qualitative Content Analysis (Mayring 2014), using inductive category formulation. This resulted in a category system that summarizes and systematizes the answers.

Confirmatory factor analyses (CFA) were performed using the lavaan package (Rosseel 2012). Different factor models for the OMQ were tested. First, a one-factor model was tested, in which all OMQ items will load on one factor. Second, the scales that have been proposed by Willis et al. (2009) were tested. This model includes an ‘openness to feedback’ factor and a ‘general attitude’ factor. Third, the two-factor model found by Smits et al. (2015) was tested with this sample. This model includes a general OMQ factor and a method factor that consists of reverse-scored items. Following the recommendations by Jackson et al. (2009), several fit measures were calculated to evaluate the different models. This includes Chi square statistics and degrees of freedom, incremental (or relative) fit measures including the TLI and the CFI, and two residuals-based (or absolute) fit measures, namely the RMSEA and SRMR. According to Hu and Bentler (1999), satisfactory model fit can be assumed if TLI and CFI > .95, RMSEA < .05 and SRMR > .09. For the normed Chi square value (x^2^/df), a value below 2 indicates good fit, while a value below 3 is acceptable (Bollen 1989). Hu and Bentler (1999) recommend using combinational rules of TLI < .95 and SRMR > .09 for sample sizes of N < 500, so these values were used for deciding on model acceptance or rejection. CFA was performed using a Robust Weighted Least Square estimation method with robust standard errors and a mean- and variance adjusted test statistic. This approach was used to replicate the findings by Smits et al. (2015), who chose this approach because of the skewness in some response distributions and the ordinal nature of their data.

## Results

### OMQ and PMQ Factor Structure

Using the survey data, various factor models that have been proposed by other authors were investigated using CFA. When cutoff values for fit measures are applied strictly, none of these proposed factor solutions could be considered satisfactory for this sample. The two-factor solution by Smits et al. (2015) reached the best fit measures. It met the criteria for ${\chi ^2}/df$, CFI and SRMR, but not for TLI and RMSEA. After inspecting factor loadings of this model, one item (No. 21) with a loading of only .09 was identified. After removing this item from the model, a TLI of .95 was reached, allowing for accepting this model under the Hu and Bentler (1999) criteria. A one factor model for the PMQ was examined. This model reached satisfactory fit measures. Table 2 summarizes the fit measures found for all tested models.

**Table 2:** Fit measures of CFA models for the OMQ scale

Model	df	${\chi ^2}$	${\chi ^2}/df$	CFI	TLI	RMSEA	SRMR
One factor	230	535.93	2.33	0.946	0.941	0.110	0.084
Willis et al. (2009)	229	522.85	2.28	0.948	0.943	0.108	0.083
Smits et al. (2015)	229	498.42	2.17	0.953	0.948	0.103	0.079
PMQ one factor	20	41.08	2.05	0.970	0.957	0.098	0.055

First three models apply to the OMQ, fourth model applies to PMQ. Scaling-corrected Chi-squared, CFI, TLI and RMSEA are reported. The TLI of the Smits et al. (2015) model was < .95 after removing item 21

### Descriptive Results

The mean OMQ score on the six-point Likert scale (1–6) in this sample was 3.656 (SD = .80). A mean sum score of 84.1 (SD = 18.39) was calculated for comparing it against the score obtained by Smits et al. (2015), who used a sum score instead of a scale mean. OMQ Scores were significantly lower than the mean score of 4.28 (SD = 0.76) reported previously by Willis et al. (2009) in their pre-training sample, d = 0.907, t(205) = 5.72, p < .0001, the mean sum score of 98.18 (SD = 11.98) by Smits et al. (2015), d = 0.696, t(275) = 7.72, p < .0001, and the mean score of 4.01 (SD = 0.56) found by Edbrooke-Childs et al. (2016) for their overall pre-training sample, d = 0.512, t(149) = 2.58, p = .011. All comparisons are statistically significant using the Bonferroni-corrected p value of .017.

Cronbach’s alpha for the total OMQ was .94, indicating very high internal consistency.

OMQ item-level data is summarized in Table 3. “Approval rates” were calculated by dividing the number of responses that at least “slightly agree” to a statement by the total number of responses. Outcome monitoring is generally viewed as useful for providing feedback based on their scores and therapists consider it valuable to develop skills in this area. However, expenditure of time seems to be an issue for a majority of participants. Regarding the intention of usage, there seems to be an ambivalence. On the one hand, less than half of the participants are confident about integrating outcome monitoring into their work or have the clear intention to offer outcome monitoring in their practice. On the other hand, the amount of therapists who try to completely avoid monitoring is comparable.

**Table 3:** OMQ item means, standard deviations and approval rates. Items are sorted by approval rate

Item	Content	Mean	SD	% Approval
17	Useful to provide feedback based on monitoring	4.261	0.970	87.39
4	Would discuss results with customer	4.486	1.285	81.08
11	There is value in developing monitoring skills	4.171	1.043	79.28
13	Measures take too long	4.018	1.221	66.67
20	Customer accepts more responsibility	3.883	1.118	66.67
3	Help motivate customers	3.874	1.192	63.96
8	Engage customers more actively	3.802	1.267	63.06
14	Clients will not mind	3.703	1.067	60.36
9	Need to develop understanding data	3.649	1.366	60.36
19	Learn more about monitoring	3.559	1.165	57.66
7	Find monitoring very useful	3.693	1.189	56.76
22	Helps treatment planning	3.532	1.256	55.86
12	Better treatment decisions	3.459	1.204	55.86
21	Don’t know how to use measures	3.676	1.287	54.05
23	Nobody has time for monitoring	3.550	1.227	53.15
16	See value in changing clinical practice	3.360	1.094	52.25
6	More collaboration between clinician and consumer	3.441	1.173	49.55
2	Confident integrating monitoring into work	3.261	1.298	47.75
18	Intention to offer monitoring to consumers	3.306	1.271	46.85
10	Avoid usage of monitoring	3.333	1.454	43.24
5	Takes human aspect out of treatment	3.306	1.278	43.24
1	Does not capture what is happening for clients	3.297	1.180	40.54
15	Questions not relevant to client	3.162	1.083	36.04

Items were abbreviated. See “Appendix 1: OMQ Items” for full item text. Scale values: 1: strongly disagree, 2: disagree, 3: slightly disagree, 4: slightly agree, 5: agree, 6: strongly agree. Approval rates were calculated by dividing the number of answers indicating approval of a statement by the total number of responses. Participants responding with 4 or higher for an item were considered “approval”. Margin of error for approval ratings: 9.25%

The mean PMQ score was 3.16 (SD = 0.96). Cronbach’s alpha for the PMQ scale was .91. OMQ and PMQ scores were correlated highly (${r_{108}}=.76,p<.001$). Item-level statistics for the PMQ are listed in Table 4. Process monitoring was considered too complex by a majority of therapists and only a small number could imagine using process monitoring applications in their practice. Also, many therapists question the validity of process monitoring.

**Table 4:** PMQ item means, standard deviations and approval ratings, i.e. percentage of participants repsonding with at least “slightly agree” to the statement

Item	Content	Mean	SD	% Approval
8	Interpretation too complex	4.225	1.263	69.37
3	Promotes self reflexion in clients	3.649	1.101	64.86
6	Processes not captured adequately by monitoring	4.009	1.247	63.96
2	Clients overcharged by monitoring	3.973	1.194	63.96
4	Unnecessary effort	3.658	1.331	55.86
1	Recognize possible deteriorations in course of treatment	3.387	1.273	54.95
5	More trust in therapeutic process	3.297	1.180	46.85
7	Could imagine using process monitoring	2.820	1.237	31.53

Items are sorted by approval rating

Items were abbreviated. See “Appendix 2: PMQ Items” for full item text. Scale values: 1: strongly disagree, 2: disagree, 3: slightly disagree, 4: slightly agree, 5: agree, 6: strongly agree. Approval rates were calculated by dividing the number of answers indicating approval of a statement by the total number of responses. Participants responding with 4 or higher for an item were considered “approval”. Margin of error for approval ratings: 9.25%

### Associations Between Therapist Characteristics and Attitudes Towards Monitoring Attitude

#### Attitude Towards Outcome Monitoring

There were no significant associations between OMQ scores and clinician age (${r_{(111)}}= - .01,\,p=.95$) or years of experience (${r_{(108)}}= - .09,\,p=.33$). Gender was not associated significantly with outcome monitoring attitude (${F_{1,95}}=3.049,\,p=.08$), while therapists reporting previous experience with monitoring systems had more a positive attitude towards outcome monitoring with a medium effect (${\omega ^2}=.033,{F_{1,95}}=5.024,\,p=.027$). Theoretical orientation significantly influenced attitudes with a medium effect as well (${\omega ^2}=.05,{F_{3,95}}=3.062,\,p=.032$). A post-hoc Tukey HSD test was conducted for the theoretical orientation ANOVA, revealing significantly better attitudes towards monitoring (p = .036) only for CBT therapists (M = 3.97, SD = 0.73) when compared to humanistic and existential therapists (M = 3.45, SD = 0.86). Cohen’s d was calculated to be − 0.642 (CI − 1.142; − 0.141) for this difference. No other orientation yielded significant differences. No significant interaction effects between variables were found. To explore whether there are differences between participants reporting monitoring experience with those who don’t an item-level comparison was done by calculating Mann–Whitney U tests. The only item showing a significant difference after Bonferroni correction for 23 comparisons was item 21 (“don’t know how to use measures”), with non-experienced participants scoring significantly higher on this item (W = 1568.5, p = .001).

#### Attitude Towards Process Monitoring

**Fig. 1:**
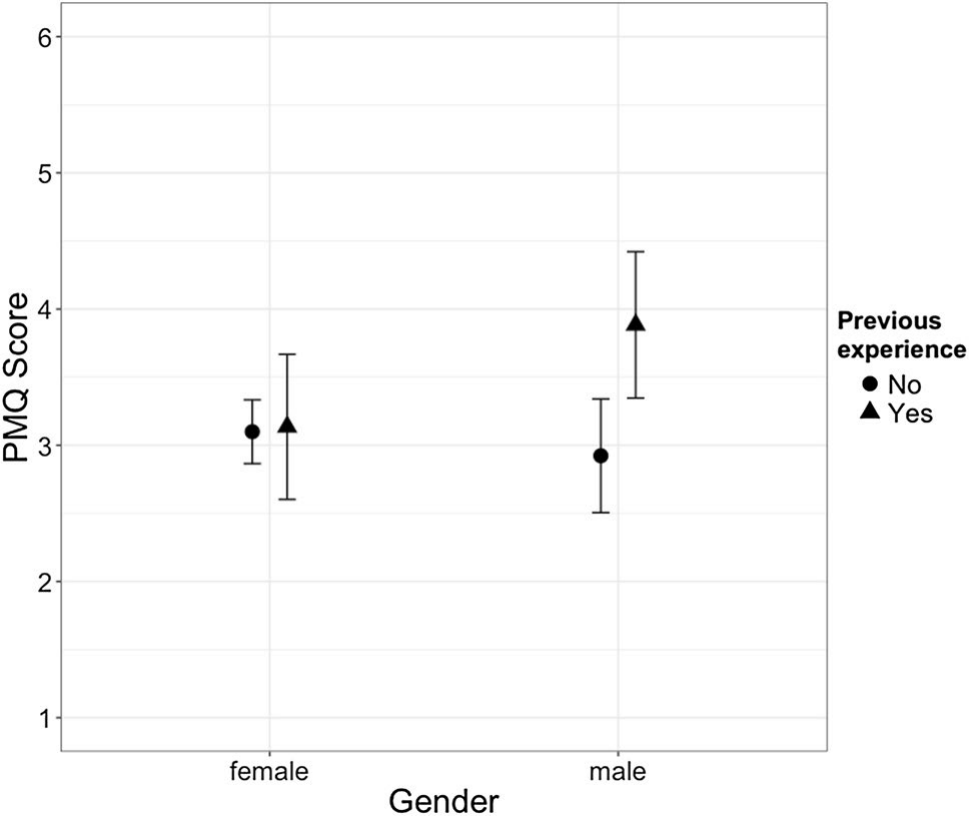
Interaction effect of clinician gender and previous monitoring experience on attitude towards process monitoring. Error bars around means indicate 95% confidence intervals

There were no significant associations between PMQ scores and clinician age (${r_{(111)}}=.03,\,p=.71$) or years of experience (${r_{(108)}}= - .08,\,p=.39$). Neither gender (${F_{1,95}}=0.839,\,p=.36$) nor theoretical orientation (${F_{3,95}}=1.185,\,p=.25$) affected process monitoring attitude. There was a significant, medium-sized effect of previous experience with monitoring systems (${\omega ^2}=.043,\,{F_{1,95}}=6.265,\,p=.014$). A significant, medium-sized interaction effect between gender and previous experience was found (${\omega ^2}=.034,\,{F_{1,95}}=5.180,\,p=.025$). A post-hoc Tukey HSD revealed that male clinicians with previous experience had significantly more positive attitudes towards process monitoring than female clinicians (p = .01) and male clinicians (p = .007) without previous experience. The interaction results are illustrated in Fig. 1. Item-wise comparisons between experienced and non-experienced therapists were conducted as well. For PMQ items, item 4 (“unnecessary effort”) was the only item showing a significant difference (W = 1531, p = .002), with non-experienced therapists scoring significantly higher on this item. Mean OMQ and PMQ scores are summarized for different genders, orientations and by previous experience with monitoring systems in Table 5.

**Table 5:** OMQ and PMQ scores (mean and standard deviation) by clinician characteristics

Characteristics	Participants	n	Mean OMQ (SD)	Mean PMQ (SD)
Gender	Women	71	3.57 (.71)	3.11 (.88)
	Men	40	3.81 (.93)	3.26 (1.09)
Previous experience with monitoring	Yes	26	3.95 (.91)	3.54 (.95)
	No	85	3.57 (.75)	3.05 (.94)
Theoretical orientation	Humanist	40	3.45 (.86)	2.95 (1.01)
	CBT	27	3.97 (.73)	3.36 (.93)
	Psychodynamic	24	3.52 (.72)	3.13 (.97)
	Systemic	20	3.82 (.75)	3.36 (.85)
	Total	111	3.66 (0.80)	3.16 (0.96)

*CBT* cognitive-behavioral therapy. Scale values: 1: strongly disagree, 2: disagree, 3: slightly disagree, 4: slightly agree, 5: agree, 6: strongly agree

### Free-Text Responses

Clinicians were able to provide arguments for and against monitoring in psychotherapy in free-text response fields. The answers were written mainly in abbreviated form or as keywords. All answers were compiled into a text file and subjected to qualitative content analysis (Mayring and Fenzl 2014) using inductive coding. Two independent raters from the research group categorized the free-text responses, resulting in two independent category systems. These category systems were then reviewed by both raters to detect disagreements in content or level of abstraction. Disagreements between those two category systems were discussed and resolved by choosing the categories that were deemed more accurate. Adequate intercoder agreement was assumed after resolving all disagreements between category systems. This way, a single category system of arguments was extracted. Possible advantages of monitoring are described in Table 6 along with an example for the respective category and their absolute frequencies. Table 7 lists all disadvantages mentioned by therapists.

**Table 6:** Therapist-identified advantages of monitoring systems and their absolute frequencies

Category	Example	Frequency
Control and visibility of changes	Accompanies therapy well, demonstrates effects of treatment	23
Improved reflection	Increases self-reflection of patients	11
Improved feedback	Offers therapist information on experiences between sessions	7
Enhanced therapy motivation	Could motivate clients to proceed with therapy	6
Objectivity, comparability	Standardization	5
Increased autonomy for patients	Increases transparency, reduces iatrogenic pathologies	4
Supporting psychotherapy research	Makes sense for research purposes	4
Increased efficiency of treatment	Increases efficiency	2

**Table 7:** Therapist-identified disadvantages of monitoring systems and their absolute frequencies

Category	Example	Frequency
Increased effort	Who is paying me for the additional work hours?	32
Negative influences on therapy	Could have negative influences on therapeutic relationship	13
Pressuring and overextending patients	Could pressure patients into providing positive results	10
Lack of validity	Disconnected from the patient’s reality	9
Deindividualization	Dehumanizing–individual is reduced to mere statistics	8
Financial efforts	Too expensive	7
Inappropriate for some patients	Additional stress for patients who tend to ruminate	7
Contradicting therapeutic style	Transference and countertransference can’t develop	4
Lack of patients’ motivation and compliance	Repetitive questionnaires getting on patient’s nerves	3
Lack of relevance of resulting data	Does not capture what is really happening in therapy	3
Problems with interpretation	Interpretation of data could be biased	3
Bureaucratization	Reduces therapist to a mere bureaucrat	3
Change of therapeutic focus	Fixation on monitoring of symptoms	3
Increased orientation towards efficiency	This implants the principle of efficiency into psychotherapy	2
Serves only reduction of treatment costs	Health insurances stop paying for therapy sessions because of early improvements	2

## Discussion

The results of the presented study suggest that the attitude towards outcome monitoring is substantially more negative among Austrian clinicians when compared to clinicians in the United Kingdom, Australia and Belgium. These findings could be explained by the fact that all other studies were mainly conducted in countries with mandatory outcome monitoring, which is likely to lead to an increased possibility of exposure to and experience with monitoring systems. This explanation is supported by the data, as previous monitoring experience was associated with a better opinion towards monitoring. Attitude towards process monitoring was slightly more negative compared to outcome monitoring. The procedures were viewed as too complex by more than two-thirds of participants, while offering relatively few benefits for treatment. There were concerns with the strain process monitoring puts on clients as well as doubts concerning its validity. Another possible explanation could be international differences in the six cultural dimensions proposed by Hofstede (2011), which can be compared using an online tool (Hofstede Insights 2017). Compared to Austria, Belgium and the United Kingdom, Austria scores lower on the “Power Distance” dimension. In Austrian culture, decentralized power structures are vastly preferred, possibly leading to a pronounced skepticism towards monitoring systems that subsume individual progress under a standardized system. Also, monitoring could be seen as a means of control exercised by institutions like mental health providers. This concern was expressed in the free-text responses as well. Future implementation efforts in Austria should consider this cultural particularity and be mindful of it. Communicating the purpose of monitoring systems as tools for helping clinicians instead of controlling them or impose standards of efficientcy on their work will be crucial.

Clinicians’ theoretical orientations accounted for a significant proportion of differences in attitudes towards outcome monitoring only. While practitioners of CBT had relatively high opinions, those following Humanistic-Existential approaches had an unfavorable attitude. One likely cause for this effect could be the vastly different views and definitions of therapy outcome among different orientations. The strong objections of Humanistic-Existential therapists concerning the measurement and study of treatment outcomes have been subject to discussion (Hoffman et al. 2012) because of their problematic implications for adapting a science-based practice approach (Green and Latchford 2012). Criticism from a Humanistic-Existential point mainly concerns definitions of treatment outcome that are seen as too narrow and overly quantitative, being mostly defined as behavioral change and symptom reduction, while being dismissive of subjective experience and positive growth. This criticism is rooted in deeper, epistemological objections to psychology as a quantitative science and its strive for the generation of “objective” explanations for psychological phenomena (Barry 1999). While this may seem to be strongly opposed to the fundamental assumptions of outcome monitoring, this strong opposition can also be seen as an argument for intensifying the dialogue between researchers and clinicians. They reflect concerns of practitioners that need to be taken seriously when implementing monitoring systems and can inform developers of future outcome monitoring instruments about possible extensions of the definition of “desirable outcomes”. Also, as Humanistic-Existential practitioners form the largest group of practitioners in Austria (see Appendix 3), excluding these practitioners would lead to the exclusion of one-third of psychotherapists. It is unlikely that these therapists will reject monitoring completely, as this would mean completely rejecting evidence on benefits for patients and the potential aid monitoring can be for therapists.

Clinicians expressed various practical advantages of monitoring systems in their free-text responses, but also a number of concerns. In the view of many clinicians, the main advantage of monitoring is increased visibility of change and increased control over the therapeutic process. Also, patients could feel more involved in their therapies by providing feedback to their therapists, their self-efficacy, self-perception and reflection could increase. Many therapists’ concerns regarding monitoring were related to problem areas also identified by Boswell et al. (2015). Administration of monitoring, scoring of questionnaires and interpretation of the data were seen as an additional time and work burden to a degree that might be bearable by larger clinics, but not for private practitioners. Also, patients might be put under stress by constant assessment routines, especially by daily assessments in process monitoring. There could also be some pressure to deliver “good” outcomes, while outcomes that are too “good” may lead health insurances to cease their payments. There were some strong objections that seem to come from criticism of psychometric measurement in general. A possible dehumanization of patients was feared by some clinicians, as both process and outcome monitoring subject a highly individual process like psychotherapy to a process of quantitative data collection and statistical analysis. This is believed to lead to abstract data sets that misrepresent the patient’s subjective experience and their particular reality. Some practitioners also feared possible negative impacts on the therapeutic relationship or interference with other relevant processes. The qualitative results are supported by quantitative data. Outcome monitoring was viewed as potentially time-consuming and the readiness to use outcome monitoring was low. A certain backlog concerning scoring and interpretation of instrument was stated as well by many participants. These results match those of a qualitative study with British therapists (Norman et al. 2014) that also identified main themes like “Implementation issues”, which included increased time and effort for already overscheduled practitioners as well as a lack of understanding of monitoring systems. Also, a main theme of “depersonalising” was identified, including objections to an overly strong focus on “numbers” to describe subjective experiences in psychotherapy.

While both questionnaires assessing monitoring attitude were reliable regarding their internal consistencies, the factor structure of the OMQ could not be replicated with satisfactory model fit. The proposed two-factor model by Smits et al. (2015) reached the best model fit indices and after removing one item, it fulfilled the criteria of good model fit. Still, further studies should seek to replicate this factor structure with larger samples and possibly participants from other German-speaking countries.

### Utility and Consequences for Policy

In their reflections on the implementation of outcome monitoring on a national level, Mellor-Clark et al. (2014) propose a schematic structure for a multiphasic implementation approach, based on the Quality Implementation Framework (QIF, Meyers et al. 2012). This study can be seen as part of the first phase of the QIF, namely host setting assessment. The results presented here are a first probe into philosophical and practical attitudes of practitioners in a country with little to no attempts of implementing monitoring in mental health, including main concerns of practitioners. To achieve this, the psychometric aspects of a German translation of the OMQ were examined using a sample of Austrian psychotherapists. Additionally, possible factors influencing monitoring attitude were studied.

Like in Germany, there is no central coordination for the implementation of outcome monitoring in Austria yet, even if future government-sponsored implementation initiatives are not out of question. Up to now, successful implementation mainly relies on bottom-up developments and the efforts of identified “local champions” (Boswell et al. 2015) or, as included in the QIF, teams of local mentors, who put great efforts into spreading information and serving as a good example when it comes to implementing monitoring systems. Similar to actions taken in the National ROM QIC, teams of local mentors identified by surveys could organize local meetings as low-threshold offers for practitioners to meet and discuss relevant aspects of monitoring in psychotherapy. Because therapists often express concern about their patients and possible negative effects of outcome monitoring on their well-being, patient representatives should be included in dialogue events. Cooperations with local stakeholders like regional healthcare providers could be promising, as many of them should be interested in improved access to psychotherapy as well as in methods of raising the effectiveness of treatments. Local academic institutions like universities, especially their departments of public health or psychology could serve as scientific bases for outcome monitoring implementation. The inclusion of patient groups will probably contribute to the success by providing other members of the initiative with their views on monitoring, hopefully serving as a counterbalance to the more policy motivated discussions that are likely to occur between researchers and practitioners. Mental health practitioners with low opinions of monitoring will hinder successful implementation, but these attitudes can be changed through such initiatives, as clearly shown by Metz et al. (2017). Another step in the National ROM QIC are training courses for the use of monitoring systems in order to learn how to use monitoring instruments and how to apply them in their therapies, including follow-up booster sessions after the more intensive trainings that can also serve as an arena of exchange of good practice.

### Limitations

The study has a number of limitations. First, a relatively low response and retention rate was achieved. With 1212 therapists contacted, only 111 (9.16%) completed the survey. Given that there were 9308 registered psychotherapists in Austria during Summer 2017 (Statistik und Daten zur Psychotherapie 2017), the sample represents 1.19% of the population. It is not uncommon for an online survey addressed to mental health professionals to reach a relatively low response rate. For example, the “2015 APA Survey of Psychological Health Service Providers” (Hamp et al. 2016) reached a 10.8% response rate for non-APA members (16.4% for members). As shown in a meta-regression by Cook et al. (2000), contact mails including personalized salutations increase the response rate of online surveys. Although this strategy was employed in the survey, the response rate remained low. Another empirically validated method is sending out follow-up mails to those who did not complete the survey. However, due to the anonymity of the web survey, it was not possible to determine the therapists who already answered, so this strategy was not feasible without contacting all 1212 therapists again. It could be argued that the response rate would have been higher if incentives were used. However, a meta-analysis of incentive effects by Göritz (2006) showed that the effect of incentives heavily depends on the baseline response rate and provided a prediction table that can be used to estimate the effect of incentives if the number of participants opening the survey URL (defined as response rate) and the number of survey completers is known. In the case of this study, the predicted increase of sample size would have been 0.84%, or ten additional subjects.

It has to be noted that the sample obtained in this study matches the population of Austrian psychotherapists quite well in terms of gender and theoretical orientation (see Appendix A1 for a comparison). The overrepresentation of cognitive-behavioral therapists in this sample could be attributed to a strategy of recruitment that addressed more CBT-oriented institutions. Given the favorable opinion of CBT therapists towards monitoring, this could have distorted our results towards a more favorable opinion. The small sample size leads to a relatively large margin of error for responses that has to be considered when interpreting the data. Because the majority of the sample has no experience with monitoring systems, most of the participants answered to a hypothetical situation based on the information provided before taking the survey. However, due to the introductory texts that every participant read, it is improbable that therapists deciding to participate did not have any notion of how monitoring systems might influence their clinical practice. Still, one has to acknowledge the limitation that this survey assumes this prior basic knowledge without checking it, so that further research should be done to examine specific differences in attitude that result from different levels of experience. Regarding the aims of this study, the inclusion of answers of therapists with no contact whatsoever during their career is important as it results in a more realistic representation of monitoring attitude in the population studied.

## Conclusion

This paper can be seen as a first contribution to a broader strategy of implementation of monitoring, because it is the first to assess the attitudes of therapists in a country with virtually no attempts of outcome monitoring implementation. Regular, quantitative assessments of monitoring attitude are a helpful tool for implementing monitoring and continuing such research may lead to valuable insights that make it possible to detect possible therapist-side obstacles in the process, as well as serve as a means of validation of attempts to increase the use of monitoring systems.

## Appendix 1: OMQ Items

### English Version

1. Outcome measures do not capture what is happening for my clients.
2. I am confident integrating outcome measures into my work.
3. Providing feedback from outcome measures will help to motivate consumers.
4. I intend to discuss the results of the consumer’s self-assessment with the consumer.
5. Outcome measures take the human aspect out of my work.
6. Providing feedback from outcome measures will help the clinician and consumer work more collaboratively in treatment.
7. I find outcome measures very useful for working with clients.
8. Providing feedback from outcome measures will help to engage consumers more actively in their own treatment.
9. I need to develop my understanding and use of normative comparison data.
10. I avoid using outcome measures as much as possible.
11. There is value in developing my skills to provide feedback on progress with consumers.
12. Using outcome measures will help me make better treatment decisions with clients.
13. Outcome measures take too long.
14. If explained properly, clients will not mind using outcome measures.
15. Most of the questions in outcome measures are not relevant to the client.
16. I see the value in changing my clinical practice to support the use of the consumer self-assessment measures.
17. It would be useful to provide consumers with feedback based on their outcome self-assessment measures.
18. I intend to routinely offer the consumer self-assessment measure to consumers.
19. I intend to learn more about outcome measures.
20. Providing feedback from outcome measures will encourage the consumer to accept more responsibility in their own treatment.
21. I do not really know how to use outcome measures to help monitor treatment progress.
22. Providing feedback from outcome measures will help with my treatment planning.
23. Nobody has time to use outcome measures routinely.

### German Translation

1. Outcome-Monitoring erfasst nicht, was bei meinen KlientInnen geschieht.
2. Ich bin zuversichtlich, Outcome-Monitoring in meine Arbeit zu integrieren.
3. Das Bereitstellen von Verlaufsfeedback an KlientInnen wird dazu beitragen, diese zu motivieren.
4. Ich habe vor, die Ergebnisse der Selbsteinschätzungen der KlientInnen mit den KlientInnen zu besprechen.
5. Outcome-Monitoring nimmt den menschlichen Aspekt aus meiner Arbeit.
6. Das Bereitstellen von Verlaufsfeedback wird dazu beitragen, dass TherapeutInnen und KlientInnen in der Behandlung mehr zusammenarbeiten.
7. Ich finde Outcome-Monitoring für die Arbeit mit KlientInnen sehr nützlich.
8. Das Bereitstellen von Verlaufsfeedback wird dazu beitragen, dass KlientInnen sich aktiver für ihre eigene Behandlung engagieren.
9. Ich muss mein Verständnis und den Gebrauch von normativen Vergleichsdaten ausbauen.
10. Ich versuche die Benutzung von Outcome-Monitoring so weit wie möglich zu vermeiden.
11. Es ist sinnvoll meine Fähigkeiten auszubauen, den KlientInnen Feedback über ihre Fortschritte zu geben.
12. Durch die Benutzung von Outcome- Monitoring werde ich bessere Behandlungsentscheidungen mit meinen KlientInnen treffen.
13. Outcome-Monitoring beansprucht zu viel Zeit.
14. Bei angemessener Erläuterung wird es KlientInnen nichts ausmachen Outcome-Monitoring zu verwenden.
15. Die meisten Fragen des Outcome- Monitorings sind für KlientInnen irrelevant.
16. Ich finde es sinnvoll, meine Arbeitsgewohnheiten zu verändern, um die Benutzung von Selbsteinschätzungsmessungen der KlientInnen zu unterstützen.
17. Es wäre sinnvoll, KlientInnen auf Basis der Ergebnisse ihrer Selbsteinschätzung Feedback zu geben.
18. Ich beabsichtige den KlientInnen Selbsteinschätzungsmessungen routinemäßig anzubieten.
19. Ich beabsichtige mehr über Outcome-Monitoring zu lernen.
20. Das Bereitstellen von Verlaufsfeedback wird KlientInnen dazu ermutigen, mehr Verantwortung für ihre eigene Behandlung zu übernehmen.
21. Ich weiß nicht wirklich, wie man Outcome-Monitoring zur Überwachung des Behandlungsfortschritts einsetzt.
22. Das Bereitstellen von Verlaufsfeedback wird mir bei der Behandlungsplanung helfen.
23. Niemand hat Zeit, um Outcome-Monitoring routinemäßig einzusetzen.

## Appendix 2: PMQ Items

### German Version

1. Mithilfe von täglichem Prozess-Monitoring kann ich mögliche Verschlechterungen im Therapieverlauf leichter erkennen.
2. KlientInnen sind mit der Technik von täglichem Prozess-Monitoring überfordert.
3. Das tägliche Prozess-Monitoring wird dazu beitragen, KlientInnen zur Selbstreflexion anzuregen.
4. Ich erachte tägliches Prozess-Monitoring als unnützen zusätzlichen Aufwand.
5. Die tägliche Auseinandersetzung mit Veränderungsprozessen im Therapieverlauf wird dazu beitragen, dass KlientInnen mehr auf den therapeutischen Prozess vertrauen.
6. Veränderungsprozesse können durch tägliches Prozess-Monitoring nicht adäquat erfasst werden.
7. Ich könnte mir vorstellen, tägliches Prozess-Monitoring in meiner Arbeit einzusetzen.
8. Die Auswertung des täglichen Prozess-Monitoring ist zu komplex, als dass ich es täglich durchführen könnte.

### English Translation

Note: this translation is for purposes of presentation only. It has not undergone a formal translation process.

1. With the help of daily process monitoring, I can recognize possible deteriorations in the treatment process more easily.
2. Clients are overstrained with the technique of daily process monitoring.
3. Daily process monitoring will promote self-reflection in clients.
4. For me, daily process monitoring poses an unnecessary additional effort.
5. Daily confrontation with change processes in the course of treatment will lead clients to trust more in the therapeutic process.
6. Change processes are not captured adequately by daily process monitoring.
7. I could imagine using daily process monitoring in my work.
8. Interpretation of daily process monitoring data is too complex for me to perform on a daily basis.

## Appendix 3

See Table 8.

**Table 8:** Comparison of sample characteristics with the general population of Austrian psychotherapists

	Sample	Population in %
N	111	8429
Gender—female (%)	64.0	72.4
Theoretical orientation		
Psychodynamic/analytic (%)	24 (21.6)	2180 (25.9)
Humanistic (%)	40 (36.4)	3159 (37.5)
Cognitive-behavioral (%)	27 (24.3)	999 (11.9)
Systemic (%)	20 (18.0)	2091 (24.7)

Population-level information taken from online database “Statistik und Daten zur Psychotherapie” (2017), including all registered psychotherapists listed with theoretical orientation
